# Influence of topography on the asymmetry of rill cross-sections in the Yuanmou dry-hot valley

**DOI:** 10.1038/s41598-022-18198-5

**Published:** 2022-08-17

**Authors:** Xingli Gu, Jun Luo, Bin Zhang, Lei Wang, Qiangjianzhong Wu

**Affiliations:** 1 School of Geographical Sciences, China West Normal University, Nanchong, China; 2 Sichuan Provincial Engineering Laboratory of Monitoring and Control for Soil Erosion in Dry Valleys, China West Normal University, Nanchong, China; 3 Liangshan Soil Erosion and Ecological Restoration in Dry Valleys Observation and Research Station, China West Normal University, Nanchong, China; 4 Institute of Geographic Sciences and Natural Resources Research, University of Chinese Academy of Sciences, Beijing, China

**Keywords:** Geomorphology, Restoration ecology

## Abstract

Rill erosion is one of the most common types of erosion, and the development conditions of the asymmetric characteristics of rill cross-sections are still relatively poorly understood. To explore the relationship between rill topography and rill cross-sectional asymmetry, we used the microtopographic profiler method to measure 712 groups of rill cross-sections in the Yuanmou dry-hot valley area. The results of correlation analysis and principal component analysis to investigate the topographical conditions of rill development show that: (1) asymmetry is the main feature in rill cross-sections; 53% of rill cross-sections are right-biased and 47% are left-biased. (2) There is an extremely significant positive correlation between the slope difference and the rill cross-section asymmetry ratio (*p* < 0.01); the asymmetry ratio increases as the slope difference on both sides (*B*) increases, and the directionality of the asymmetry ratio is affected by *B*. The difference between the catchment areas on both sides has a significant linear correlation with the asymmetry ratio of the width (r = 0.07, *p* < 0.05). (3) Seven topographic factors were divided into two types of principal components: the first represents the rill slope surface shape and the rill shape, and the second represents the difference between the two sides of the rill.

## Introduction

Soil erosion is one of the main environmental problems affecting humans and leads to around 5 to 7 million hectares of farmland loss every year^1^. Gully erosion is one of the most important soil erosion processes and results in a soil loss rate of around 85%^2^. Rill erosion is one of the initial forms of channel erosion^3^, usually eventually forming a gully^4^. Research on rills has mainly focused on the origin of rill cross-sectional morphologies^5^, and describing the relationship between general rill cross-sectional morphology and rill erosion^6–9^. The morphological characteristics of rills form the basis for understanding the underlying mechanisms of the evolution of rills and are important for estimating rill erosion volumes and rates^10–12^. Therefore, it is paramount to study the morphological characteristics of rill erosion.

Rills can exhibit a planar, cross-sectional, or longitudinal morphology, and the cross-section is the most important morphological feature reflecting the development stage of the rill^13^. In the early stages of rill development, the rill cross-section generally presents a “V” shape^14^. With the continuous evolution of a rill, a “U” or “box” cross-section gradually appears, but the rill is still dominated by a “V”-shaped cross-section^15,16^. The morphological characteristics of a rill cross-section include its length^17^, depth^17^, width^18^, and asymmetry ratio^19^, and these characteristics change with the evolution of the rill^14^. The length of a rill has a significant positive correlation with the evolution rate of its morphology, and its width and depth increase with length^18^. However, an increase in rill length and width leads to an increase in runoff and rill depth, and rill erosion becomes increasingly intense, which accelerates rill development^17,20^. The cross-sectional area of a rill is positively correlated with the rill catchment area. The greater the flow of water into a ditch, the more serious the rill erosion, and the larger the rill cross-sectional area^21^. Asymmetry in a cross-section is the main feature in eroding gullies and it is an extremely important parameter used to describe the morphological and dynamic characteristics of a watershed^22^. Cross-sectional asymmetry was first used to evaluate the morphologies of river beds and then to describe the morphologies of channels^23^. However, a method for the systematic quantification of rill asymmetry is still lacking.

Cross-sectional asymmetry is the result of several combined factors, such as bedrock^24,25^, climate^26^, vegetation^27,28^, and topography. With regard to bedrock, studies in loess areas have revealed that a cross-slope section is steeper on the exposed side of the bedrock and gentler on the side covered by the loess layer. This is because it is more difficult to erode bedrock than to erode loess deposits^25^. Regarding climate, the sunward side is easily corroded by glacial melt runoff and expands to the rear side to form an asymmetric channel^26^. Indeed, when the solar incident angle is high in the afternoon and at noon, the temperature of the groove wall on the sunward side rises, making it more susceptible to erosion^26^. For vegetation, a slope with good vegetation development has relatively low levels of runoff and erosion; a slope with poor vegetation development, or bare patches, has relatively high levels of runoff and erosion. The varying levels of runoff and erosion are integral in forming an asymmetrical channel cross-section^27,28^. Finally, differences in topographic factors, such as slope^29^, slope length^29^, gully depth^30^, and catchment area, all affect the evolution of asymmetrical cross-sections. Scholars currently believe that topography indirectly influences small regional climates and vegetation conditions, resulting in channel cross-sectional asymmetry. Most studies have focused on large-scale channels like gullies, with few studies considering the rill cross-sectional asymmetry ratio ^24,25,27,31^.

Several studies on rill cross-sectional asymmetry, the influence of cross-sectional asymmetry on rill erosion, and the causes of cross-sectional asymmetry have been conducted. However, quantitative research on the asymmetry characteristics of rill cross-sections is still lacking, and how topographic factors affect rill cross-sectional asymmetry remains unresolved. The objectives of this study are: (1) to establish a rill cross-sectional asymmetric morphology index that describes the rill’s cross-sectional shape and permits the selection of key topographic factors; and (2) to investigate the relationship between rill cross-sectional asymmetry and rill topographic factors. This reveals the evolutionary laws and mechanisms underlying rill morphology and provides a reference for ecological restoration and soil erosion management.

## Results

### Statistical characteristics of rill cross-sectional asymmetry (RCA)

**Figure 1:**
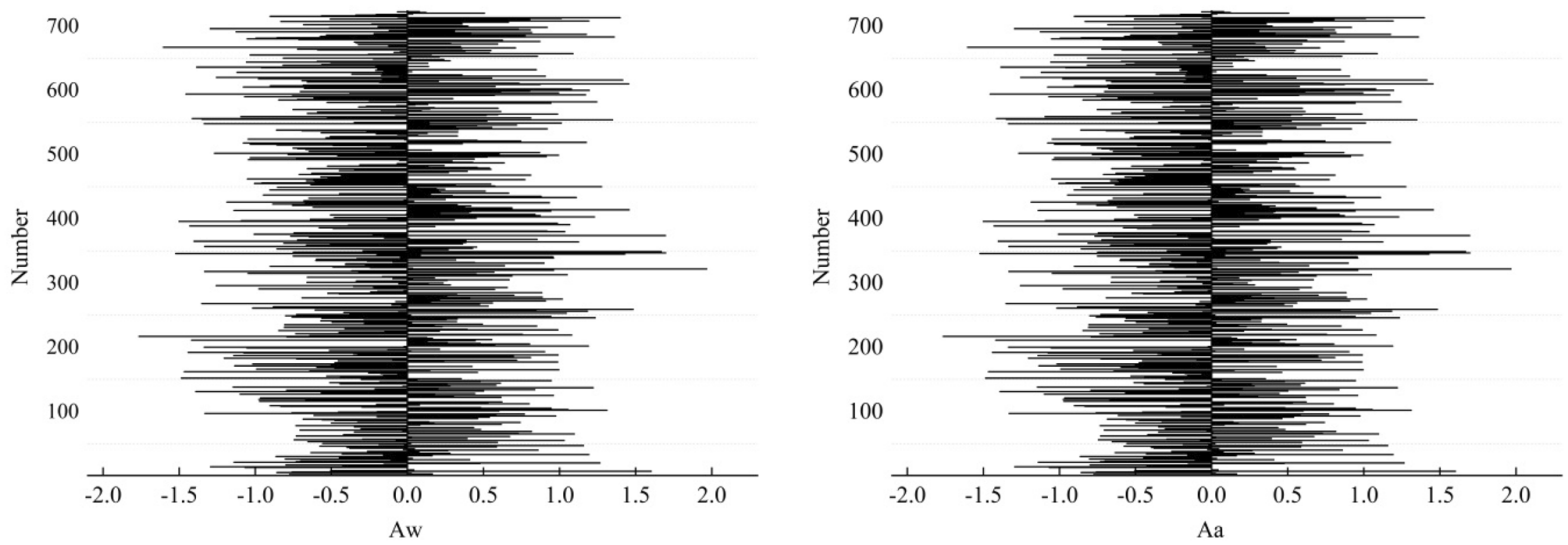
Statistical characteristics of the rill cross-sectional asymmetry (RCA).

The rill cross-sectional asymmetry (RCA) is a key parameter in describing rill morphology and includes the asymmetry ratio of the width (*Aw*) and the asymmetry ratio of the area (*Aa*). It reflects the differences in certain aspects of natural conditions resulting in inconsistent development speeds on both sides of a rill cross-section. The cross-section was classified as left-biased if *Aw*, *Aa* < 0, quasi-symmetrical if *Aw*, *Aa* = 0, and right skewed if *Aw*, *Aa* > 0. The left/right deflection reflects that erosion on the right happened faster than on the left, so the slope on the left is not as steep as on the right. The results of this study show that asymmetry is a common phenomenon in the cross-section of a rill. The *Aw* ranged from − 1.77 to 1.97, with an average value of − 0.034. There were 374 cross-sections whose RCA was less than or equal to 0, meaning that 53% of the cross-sections were right-biased. The *Aa* ranged from − 1.81 to 1.71, with an average of − 0.046. There were 374 cross-sections with an RCA of less than or equal to 0, meaning that 53% of the cross-sections were right-biased (Fig. 1).

**Figure 2:**
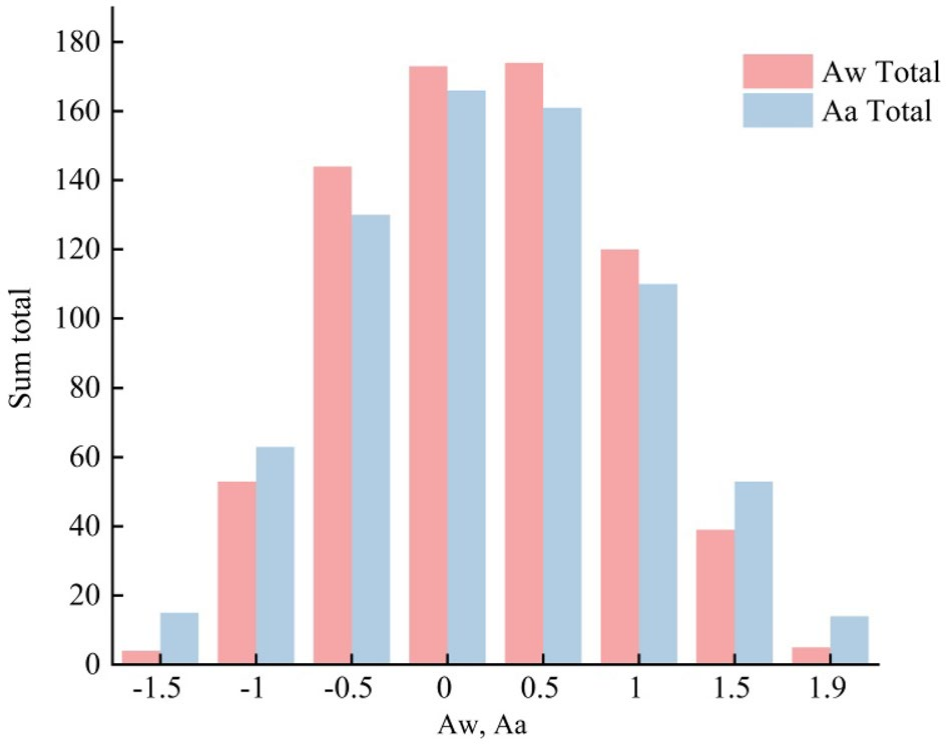
Distribution characteristics of the RCA.

Figure 2 shows that there are four *Aw* groups in the interval (− 1.7, − 1.5), 53 groups in the interval (− 1.5, − 1.0), 144 groups in the interval (− 1.0, − 0.5), 173 groups in the interval (− 0.5, 0), 174 groups in the interval (0, 0.5), 120 groups in the interval (0.5, 1.0), 39 groups in the interval (1.0, 1.5), and five groups in the interval (1.5, 2). The *Aa* has 15 groups in the interval (− 1.8, − 1.5), 63 groups in the interval (− 1.5, − 1.0), 130 groups in the interval (− 1.0, − 0.5), 166 groups in the interval (− 0.5, 0), 161 groups in the interval (0, 0.5), 110 groups in the interval (0.5, 1.0), 53 groups in the interval (1.0, 1.5), and 14 groups in the interval (1.5, 2). The RCA of most cross-sections is concentrated in the interval (− 0.5, 0.5). This interval of *Aw* contains 491 cross-sections, accounting for 68.96% of the total. There are 470 cross-sections in this interval of *Aa*, accounting for 66.01% of the total. This indicates that, although the rill cross-section exhibits some asymmetry, the difference between both sides of the section is small (Fig. 2).

### The influence of a single topographic factor on the RCA

**Figure 3:**
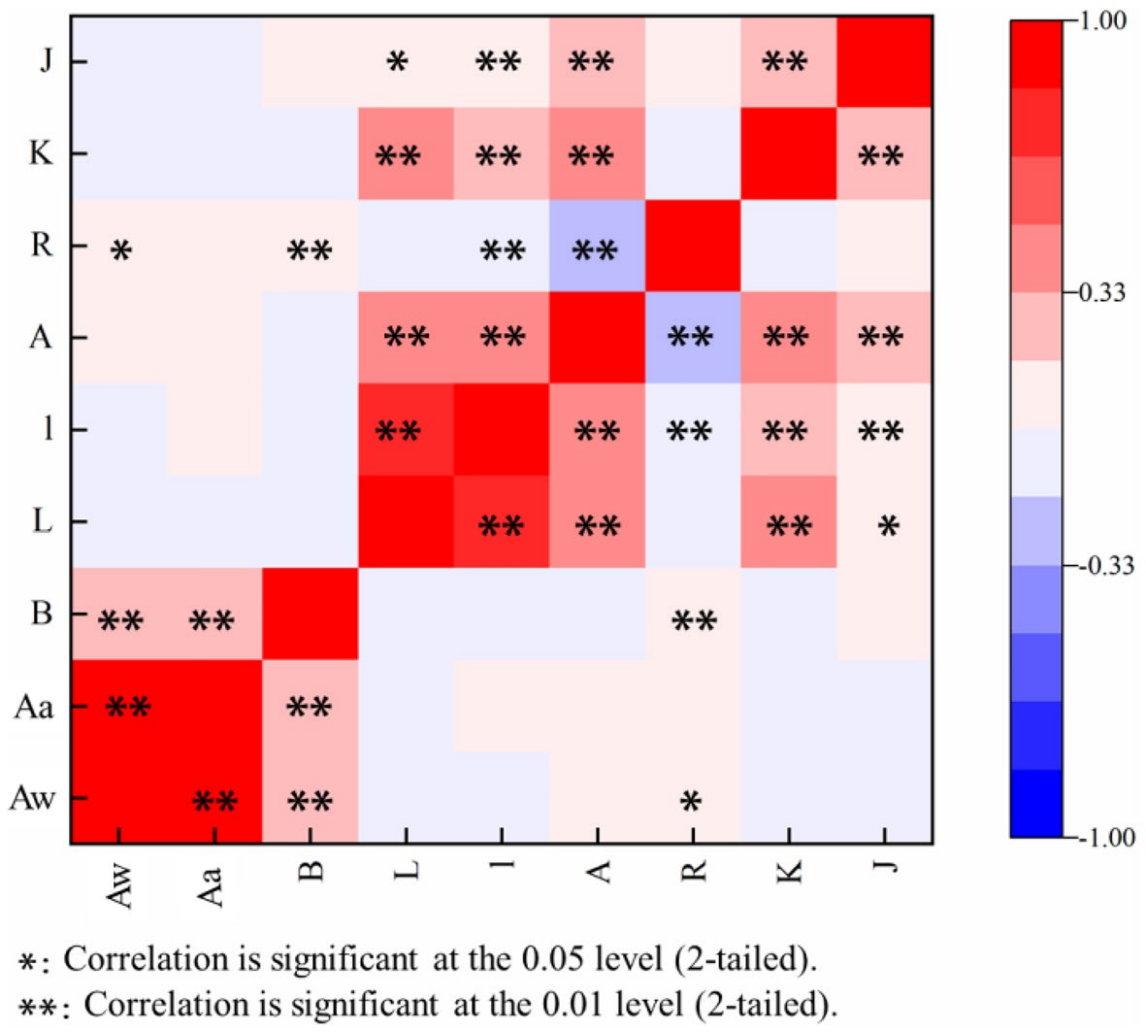
Correlation between rill cross-sectional asymmetry (RCA) and topographic factors.

Correlation analyses of the *Aw*, *Aa*, and the slope difference on both sides (*B*), rill length (*L*), rill slope length (*I*), rill head catchment area (*A*), difference between the catchment areas of both sides (*R*), rill bending coefficient (*K*), and location of the section angle of turning of the rill (*J*) were carried out. The results show that the main factors that have a significant linear correlation with the *Aw* and the *Aa* are *B* (*p* < 0.01), with correlation coefficients of 0.32 and 0.22, respectively (Fig. 3). That is, the greater the difference in slope between the two sides, the more asymmetric the rill cross-section. *R* also has a significant linear correlation with the *Aw* (*p* < 0.05), with a correlation coefficient of 0.07. This means that the greater the difference in the catchments between the left and right sides of the rill, the greater the asymmetry of the rill cross-section. However, other topographic factors have no significant correlation with the RCA.

**Figure 4:**
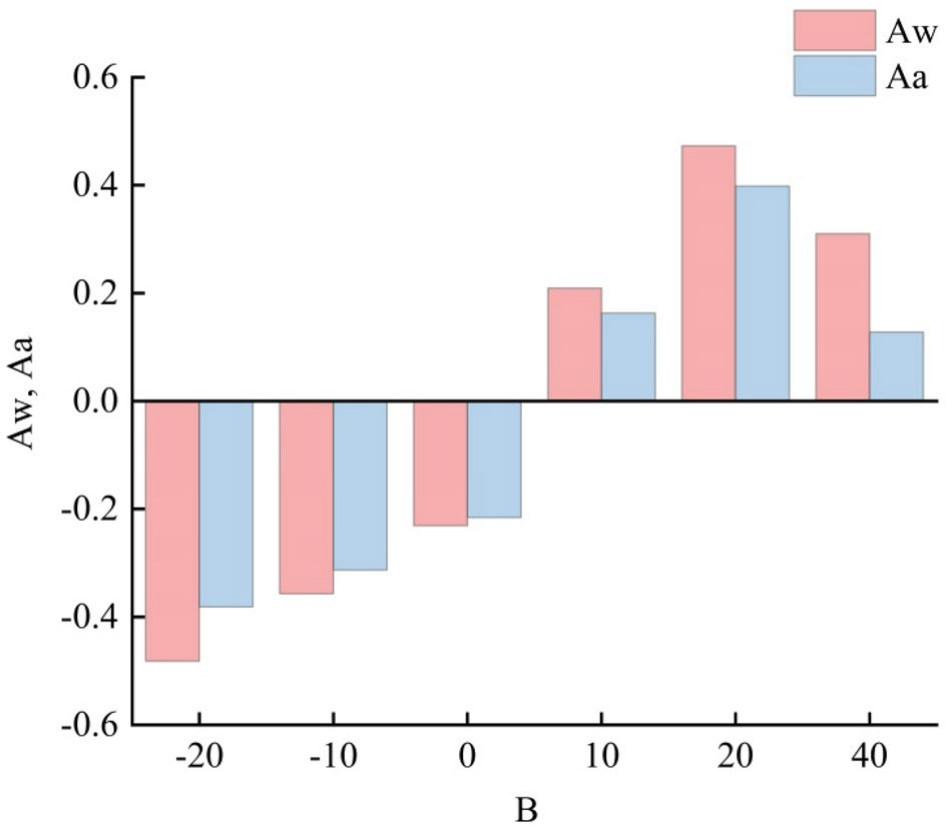
The asymmetry of different *B* values.

*B* is the difference in slope between the left and right sides of the rill cross-section catchment area. The closer *B* gets to 0, the smaller the difference in slope between the left and right sides of the rill cross-section catchment area. When the catchment area slope on the right side of the cross-section is greater than that on the left side, *B* < 0; and when the catchment area slope on the left side of the cross-section is greater than that on the right side, *B* > 0. Grouping *B* reveals that the average RCA increases as *B* increases (Fig. 4). When *B* is (− 30, − 20), *Aw* is − 0.48 and *Aa* is − 0.38; when *B* is (− 20, − 10), *Aw* is − 0.36 and *Aa* is − 0.31; when *B* is (− 10, 0), *Aw* is − 0.23 and *Aa* is − 0.22; when *B* is (0, 10), *Aw* is 0.21 and *Aa* is 0.16; when *B* is (10, 20), *Aw* is 0.47 and *Aa* is 0.40; and when *B* is (20, 40), *Aw* is 0.31 and *Aa* is 0.13. These are relatively low values because this group only has two sets of cross-sections which cannot represent the characteristics of interval *B*. The sign of the RCA is the same as the sign of *B*. The directionality of the RCA is significantly affected by *B*. When the slope of the left catchment area is large, RCA > 0, and the rill cross-section appears to be left-biased; when the slope of the right catchment area is large, RCA < 0, and the cross-section appears to the righ-biased.

### The influence of multiple topographic factors on the RCA

In order to explore the influence of multiple topographic factors on the RCA, principal component analysis (PCA) was used to extract the main feature components of the topographic data. The PCA results show that the nine topographic factors can be reflected by two principal components at 61.84% (characteristic value: 3.117+1.211=4.328 variables) (Table 1). Therefore, the analysis of the first two principal components could reflect most of the information from all the data.

**Table 1:** Calculation results of topographic factor principal component analysis (PCA).

Component	1	2
Total	3.117	1.211
% of Variance	44.534	17.303
Cumulative %	44.534	61.838
*B*	− 0.238	0.689
*L*	0.885	0.163
*I*	0.811	0.241
*A*	0.875	− 0.003
*R*	− 0.587	0.557
*K*	0.602	− 0.111
*J*	0.384	0.574

The contribution rate of the first principal component is 44.534%. The characteristic is that the factor variables have high positive loads for the four factors *L*, *I*, *A*, and *K*. *L* has the largest contribution rate at 88.5%, followed by *A*, *I*, and *K*, at 87.5%, 81.1%, and 60.2%, respectively. Therefore, the first component represents the rill slope and rill shape.

The contribution rate of the second principal component is 17.303%. The characteristic is that the factor variables have high positive loads for the three factors *B*, *J*, and *R*. *B* has the largest contribution rate at 83.5%, followed by *J* and *R*, at 57.4% and 55.7%, respectively. Therefore, the second component represents the effect of the difference between the two sides of the rill.

Based on the correlation between the topographic factors and the RCA of a rill cross-section in the Yuanmou dry-hot valley area, the following was observed: asymmetry in rill cross-sections is ubiquitous. The distribution range of *Aw* is − 1.77 to 1.97, the average value is − 0.034, and the cross-section that is right-biased accounts for 53%. A correlation analysis of the RCA and seven topographic factors shows that *B* has a significant positive correlation with the *Aw* and *Aa* (*p* < 0.01), the average RCA increases as *B* increases, and the directionality of the RCA is affected by *B*. When *B* > 0, RCA > 0, and the rill cross-section appears to the left; when *B* < 0, RCA < 0, and the cross-section appears to the right. The difference in catchment area between the sides has a significant linear correlation with the *Aw* (*p* < 0.05). Other single topographic factors have no significant correlation with the RCA. Principal component analysis and calculations show that the first principal component represents the influence of the rill slope surface and rill shape on the rill cross-sectional asymmetry. The contribution rate is 44.534%, which is characterized by a high positive load on the *L*, *I*, *A*, and *K* factors. The second principal component represents the effect of the difference between the two sides of the rill. The contribution rate is 44.534%, which is characterized by a high positive load on the *B*, *J*, and *R* factors.

## Discussion

### Influencing factors of rill asymmetry

This study shows that the difference in slope between the left and right sides of a rill has an extremely significant positive correlation (*p* < 0.01) with the *Aw* and the *Aa* and the difference in rill catchment area between both sides has a significant linear correlation (*p* < 0.05) with the *Aw*. Other single topographic factors have no significant correlation with the RCA but instead, these topographic factors have a significant correlation among themselves. They jointly affect the RCA, thus showing a high degree of correlation. Regarding the influence of multiple topographical factors on rill cross-sectional asymmetry, the first principal component represents the rill slope and rill shape, including factors *L*, *I*, *A*, and *K*. The second component represents the effect of the difference between the two sides of the rill, including the *B*, *J*, and *R* factors.

The cross-sectional shape of the rill is influenced by a variety of factors, including rainfall, vegetation, soil, runoff, slope features, and human activities, in addition to topographical factors. Vertical rainfall splashing on the slope will lead to rill erosion on it^32^. The degree of rill erosion varies with the intensity of rainfall^33^. After rainfall produces runoff on the slope, it erodes the rill slope. The greater the runoff on the slope, the greater the amount of rill erosion^34^. The degree of surface vegetation coverage is an important indicator that determines the erosion resistance of slope rills. In fact, vegetation has the potential to lessen the immediate impact of rainfall on the surface as well as the severity of runoff erosion^35^. Soil erosion resistance is also a main factor affecting rill erosion. Soils with greater clay content appear to form narrower and deeper rills for a given erosive force^36^. The evolution of rill cross-sections is not only affected by natural conditions but also by human activity. Both are important in shaping the rill morphology and accelerating soil erosion^37^. Harmful farming methods enhance rill erosion in locations with severe rill erosion, which leads to faster soil erosion on slopes. As a result, human impacts are a crucial factor that cannot be overlooked.

### The directionality of RCA

The results show that the asymmetry of rills is indeed widespread. Since the RCA is affected by many factors, such as rainfall, vegetation, soil, runoff, slope features, and human activities, some subtle differences on both sides of the rill will contribute to the asymmetry of the cross-sectional shape of the rill. The RCA of most cross-sections is concentrated in the interval (− 0.5, 0.5), accounting for 68.96% of the *Aw*’s total,and 66.01% of the *Aw*’s total, implying that the difference between both sides of the section is slight. This may be due to the small scale of the rill and small variations in natural conditions, such as topography, on both sides of the section. The results for the morphological characteristics of gully cross-sections in the Yuanmou dry-hot valley show that 385 out of 456 cross-sections measured were asymmetric in the study area^19^. Previous researchers have found that as the gully order increases in the Loess Plateaus, the degree of gully asymmetry weakens gradually due to an evident scaling effect in the gully asymmetry expression^25^. Areal asymmetry indices obtained from studies in India have also shown that lower-order and higher-order gullies are more symmetrical, while intermediate-order gullies depict a relatively higher asymmetry^38^.

**Figure 5:**
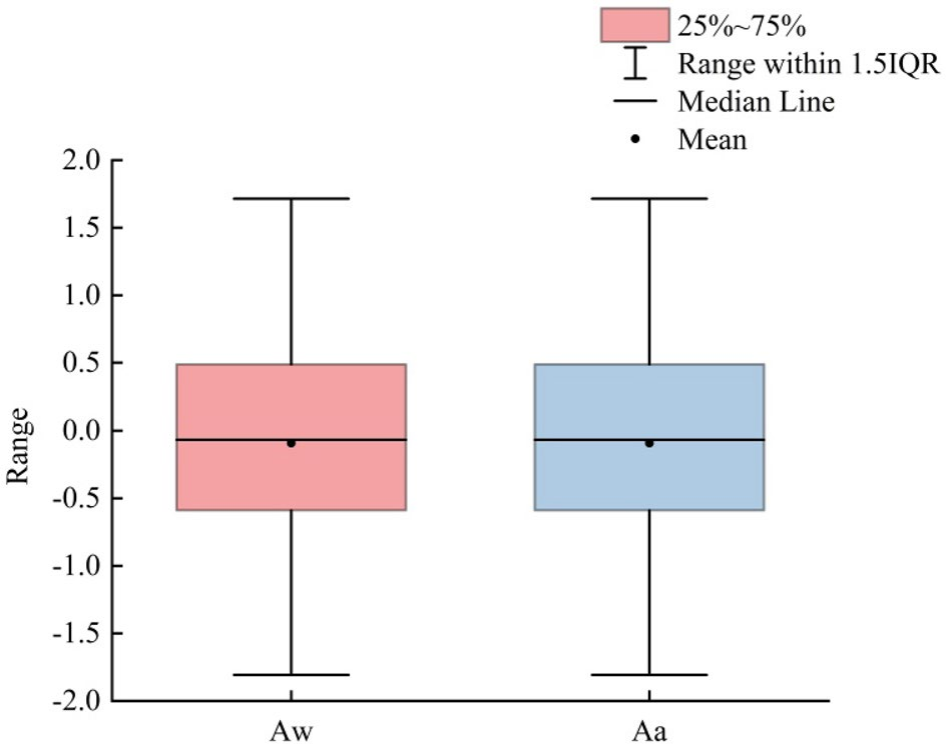
Directionality of the rill cross-sections.

Out of the 712 cross-sections studied, 53% were right-biased. This means that most of the rills were shifting to the right (Fig. 5). This is consistent with the results for the morphological characteristics of gully cross-sections in the Yuanmou dry-hot valley. Results have shown that of all the 456 cross-sections analysed in the study area, 201 were right-biased, 184 were left-biased, and 71 were quasi-symmetrical^19^. Moreover, statistical results for loess gullies have shown that most watersheds shift towards the right of the geometric center line, thereby forming a specific asymmetrical gully morphology^25^.

This study shows that the left-right bias of the RCA is consistent with *B*, and that *B* has a certain effect on the direction of the RCA. In the Northern Hemisphere, when water flows from upstream to downstream, it is deflected to the right under the action of the Coriolis force; this causes more serious erosion on the right side of a rill. However, the Coriolis force may be too small to directly affect the asymmetry of a cross-section^19^. However, many factors, such as rainfall, vegetation, soil properties, topography, and human activity, affect rill morphology^25^. The influence of such factors on rill morphology requires further study, especially to determine what causes right deviation and left deviation from a channel cross-section.

## Methods

### Study area

**Figure 6:**
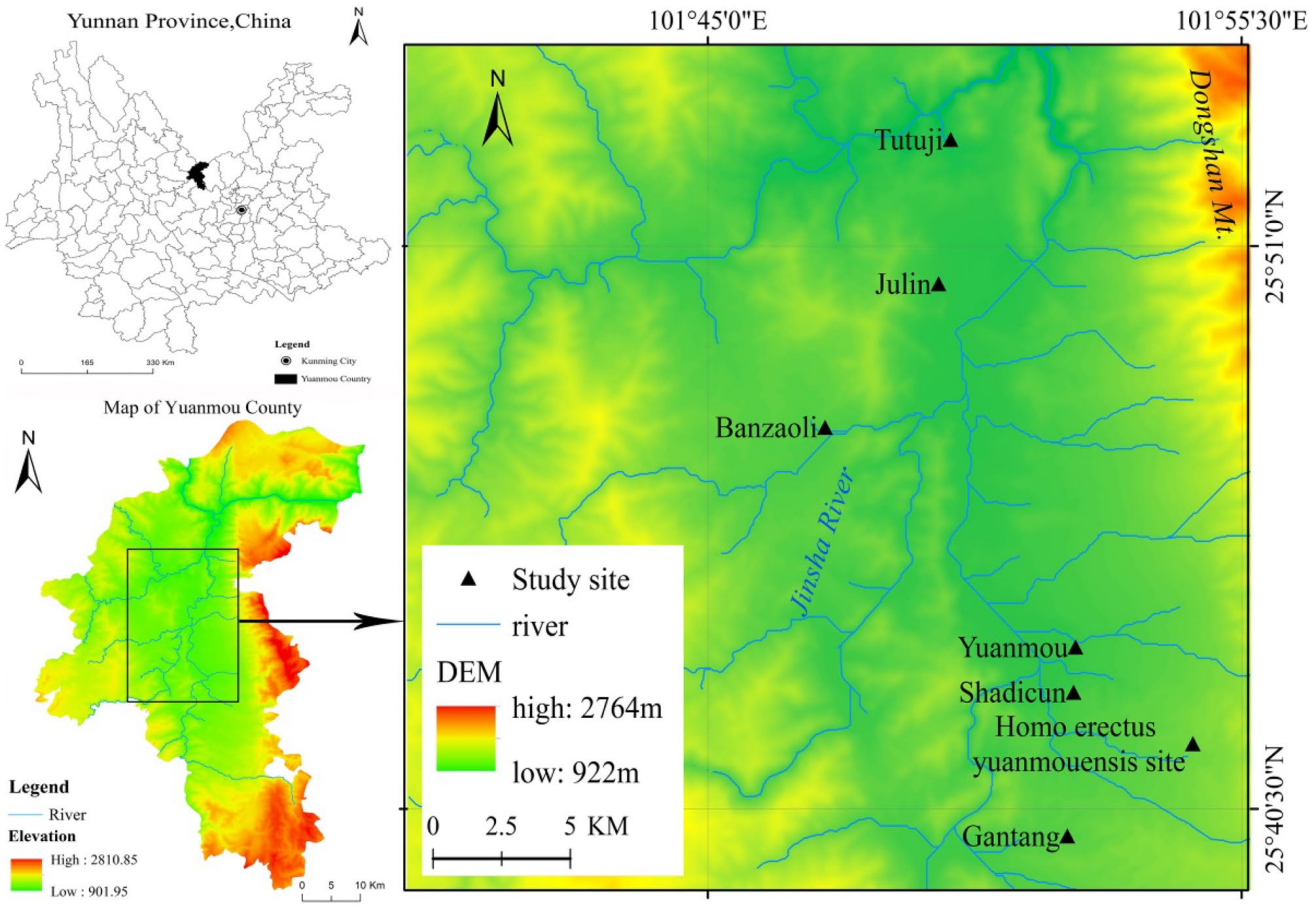
Location of the study area. Note: This map was created using ArcGIS Desktop 10.8.1 (ArcMap) software: https://www.esri.com/en-us/arcgis/products/arcgis-desktop/overview.

Yuanmou dry-hot valley is located in the northern part of Yunnan Province (101˚42′–102˚09′E, 25°33′–26°24′N). In the eastern part of the valley is situated Dongshan Mountain, with an altitude of more than 3700 m. The western part is a gentle slope. Most of the exposed rock layers in the area consist of metamorphic rocks, sandstones, mudstones, and mid-late Pleistocene terrace deposits. Fluvial and lacustrine sedimentary rocks are widely distributed at the bottom of the Yuanmou Basin^39^. The climate in the study area is dry and hot, with an average annual temperature of 21.9°C, long periods of sunshine throughout the year, an average annual rainfall of 630 mm, 5.9 times of the average annual precipitation. The zonal soils contain dry red soils, vertisols, and alluvial soils^40^. The vertisols and the alluvial soils have very poor anti-erosion capacity^39^. In addition, the vegetation coverage on the surface is very low. Considerable rainfall occurs during the rainy season (June to October), which makes the soil erosion in this area extremely serious. This soil erosion greatly restricts the local economic development and human safety^41^. The Shadi Village, Gantang, Tutujiliangzi, Yuanmouren Site, Julin, and Banzaoli Village were chosen as the study areas; the soils in all these areas consist of vertisols. Gully terrains in the region are extensively developed due to the special geographical environment and the interference of human activities. Indeed, gully erosion in these areas is very serious and the topography is broken (Fig. 6).

### Data acquisition

**Figure 7:**
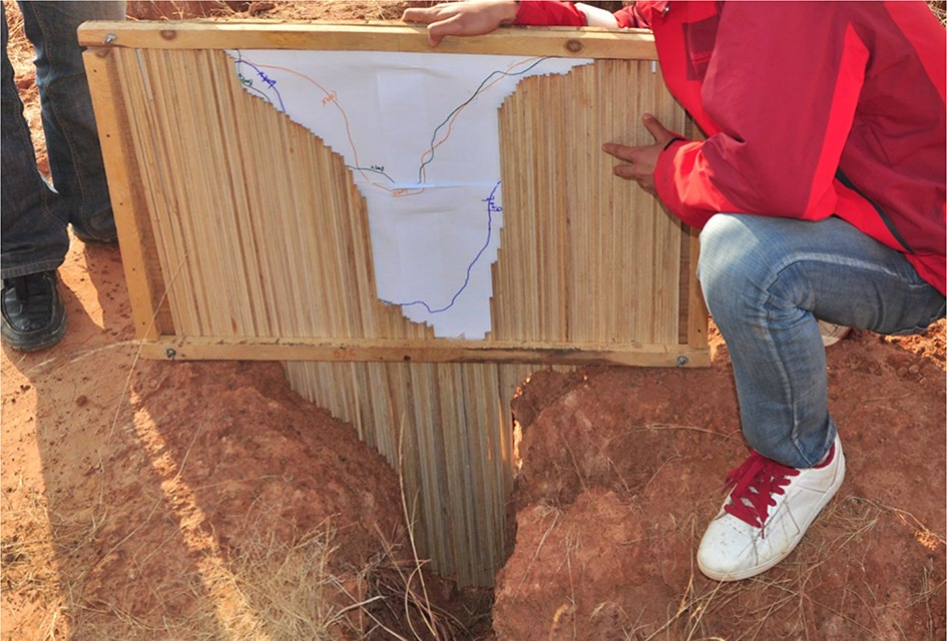
The microtopographic profiler.

One hundred and sixty-six rills were randomly selected in the study area. According to the different rill lengths, three to five typical cross-sections were selected at equal intervals for surveying and mapping, at the head, upper middle, lower middle, and upper parts of the grooves, respectively. A total of 712 cross-sections were acquired, and a microtopographic profiler method was used to trace the selected rill cross-sections (Fig. 7). A dinometer was used to measure the slope of the trench wall on both sides of the trench at the location of the section. The rill slopes were measured on both sides of the rills at the section’s position. The length of the rill slope, the actual length of the rill, the straight line length of the rill, and the height difference of the rill developed slope surface were measured with a tape measure, and the catchment areas on the left and right sides of the section and the catchment area at the head of the rill were calculated. A compass was used to measure the overall direction of the rill and the direction of the upper and lower sections, and the turning angle of the rill at each section was calculated.

## Methods

### Rill cross-sectional parameters

**Figure 8:**
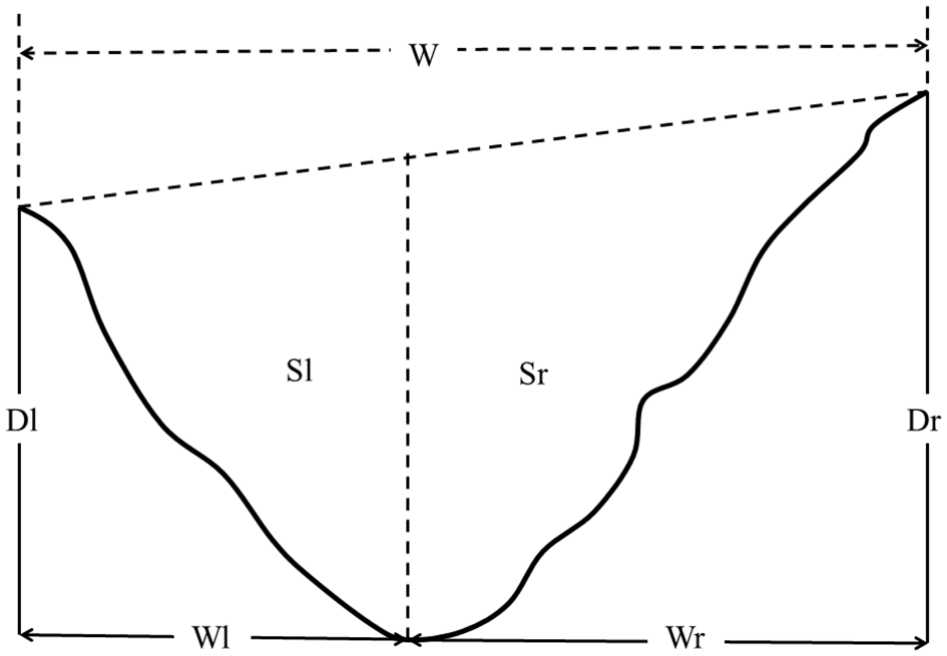
Schematic diagram of cross-sectional parameters.

Data processing comprised the following: the measured cross-sectional data was corrected using a coordinate conversion formula, and the AutoCAD2018 software was then used to extract the cross-sectional morphological parameters. The general morphological indicators of a rill cross-section were measured, namely the width (*W*), rill depth (*D*), actual rill length (*L*), asymmetry ratio of width (*Aw*), and asymmetry ratio of area (*Aa*)^19^. Details of the rill cross-sectional parameters are shown in Fig. 8 and Table 2.

**Table 2:** Cross-sectional parameters of a rill.

Parameter		Definition, formula, and significance
*Wr*	right width	Horizontal distance between the right vertex and the bottom
*Wl*	left width	Horizontal distance between the left vertex and the bottom
*Dr*	depth of right side	Vertical distance between the right-top and the bottom
*Dl*	depth of left side	Vertical distance between the left-top and the bottom
*Sr*	area of right side	Eroded area of left side
*Sl*	area of left side	Eroded area of right side
*S*	area of cross-section	*S* = *Sl* + *Sr*, describes the total eroded area
*Aw*	asymmetry ratio of width	*Aw* = (*Wl*-*Wr*)/[ (*Wl* + *Wr*)/2], describes the difference in distance between the right and left erosion
*Aa*	asymmetry ratio of area	*Aa* = (*Sl*-*Sr*)]/[ (*Sl* + *Sr*)/2], describes the area difference between right and left erosion

Revised by parameters in Deng's research^19^.

### Topographic factors

Microsoft Excel was used to calculate the slope difference between the left and right sides of the rill wall, the length of the rill slope, the catchment area of the rill head, the difference between the left catchment area and the right catchment area, the bending coefficient of the rill, and the cross-sectional position turning angle. Details of the parameters of the topographic factors are shown in Table 3.

**Table 3:** Topographic parameters.

Parameter		Definition, formula, and significance
*Bl*	Left-side slope	Slope of the catchment area to the left of the rill cross-section
*Br*	Right-side slope	Slope of the catchment area to the right of the rill cross-section
*B*	Slope difference on both sides	*B* = *Bl*-*Br*, describes the difference in slope between both sides of the catchment area to the rill cross-section
*L*	Rill length	Distance from the head of the rill to the tail of the rill
*I*	Rill slope length	Length of the slope where the rill is located
*A*	Rill head catchment area	
*Rl*	Catchment area on the left side of the section	Area enclosed from the left side of the section to below the trench head or the previous section
*Rr*	Catchment area on the right side of the section	Area enclosed from the right side of the section to below the trench head or the previous section
*R*	Difference between the catchment areas of both sides	*R* = *Rl*-*Rr*, describes the difference in catchment area between both sides of the rill section
*K*	Rill bending coefficient	Ratio of the trench bottom curve length to the trench bottom straight line length
*J*	Location of section angle of turning of rill	Difference between the inflow direction of the upper rill water and the outflow direction of the lower rill water at the section location

Revised by parameters in Deng's research^19^.

## Analysis

### Simple correlation analysis

Simple correlation analysis is a statistical analysis method used to study the correlation between two or more random variables for a given position. It can effectively indicate whether two variables change in the same direction or in the opposite direction. The simple correlation coefficient calculation formula is^42^:

1 $$R_{xy} = \frac{{\sum\limits_{i = 1}^{n} {(x_{i} - \overline{x})(y_{i} - \overline{y})} }}{{\sqrt {\sum\limits_{i = 1}^{n} {(x_{i} - \overline{x})^{2} } } \sqrt {\sum\limits_{i = 1}^{n} {(y_{i} - \overline{y})^{2} } } }}$$

where *R*_*xy*_ represents the simple linear correlation coefficient between the *x* and *y* influencing factors, *x*_*i*_*, y*_*i*_ represent the related parameters of the rill cross-sectional and topographic factors. The value range of *R*_*xy*_ is (− 1, 1). When *R*_*xy*_ < 0, there is a negative correlation between the two influencing factors, and when *R*_*xy*_ > 0, there is a negative correlation.

### Principal component analysis

This literacy uses factor analysis to analyze the topographic factors and RCA with descriptive statistics in the Statistical Package for Social Science (SPSS) Version 20 software. There was a strong correlation between the topographic factors and the concomitancy probability of Bartlett’s Test of Sphericity was 0, i.e., less than the significance level of 0.05. Therefore, the datasets in this study were suitable for factor analyses (Table 4).

**Table 4:** Kaiser–Meyer–Olkin (KMO) and Bartlett’s test.

Kaiser–Meyer–Olkin measure of sampling adequacy		0.644
Bartlett’s Test of Sphericity	Approx. Chi-Square	349.530
	df	21
	Sig	0.00X

## Supplementary Information

Supplementary Information.

## Data Availability

The datasets used and/or analysed during the current study available from the corresponding author on reasonable request.
